# A Hybrid Sensor Fault Diagnosis for Maintenance in Railway Traction Drives

**DOI:** 10.3390/s20040962

**Published:** 2020-02-11

**Authors:** Fernando Garramiola, Javier Poza, Patxi Madina, Jon del Olmo, Gaizka Ugalde

**Affiliations:** Faculty of Engineering, Mondragon Unibertsitatea, 20500 Arrasate-Mondragón, Spain; jpoza@mondragon.edu (J.P.); pmadina@mondragon.edu (P.M.); jdelolmo@mondragon.edu (J.d.O.); gugalde@mondragon.edu (G.U.)

**Keywords:** fault diagnosis, railway, model-based approach, data-driven approach, sliding mode observer, sensor fault reconstruction, condition-based maintenance

## Abstract

Due to the importance of sensors in railway traction drives availability, sensor fault diagnosis has become a key point in order to move from preventive maintenance to condition-based maintenance. Most research works are limited to sensor fault detection and isolation, but only a few of them analyze the types of sensor faults, such as offset or gain, with the aim of reconfiguring the sensor in order to implement a fault tolerant system. This article is based on a fusion of model-based and data-driven techniques. First, an observer-based approach, using a Sliding Mode observer, is utilized for sensor fault reconstruction in real time. Then, once the fault is detected, a time window of sensor measurements and sensor fault reconstruction is sent to the remote maintenance center for fault evaluation. Finally, an offline processing is carried out to discriminate between gain and offset sensor faults, in order to get a maintenance decision-making to reconfigure the sensor during the next train stop. Fault classification is done by means of histograms and statistics. The technique here proposed is applied to the DC-link voltage sensor in a railway traction drive and is validated in a hardware-in-the-loop platform.

## 1. Introduction

During the last decade, maintenance in railway systems has started moving from corrective and time-based preventive maintenance to condition-based maintenance (CBM). The CBM framework can be set based on the ISO standard 13374 [1]. L Mortellec presented a proposition for ISO 13374 implementation in mobile systems as trains [2]. Different architectures are shown, depending on the location of diagnosis, remote or on-board, and the type of diagnosis, centralized or decentralized. 

**Figure 1:**
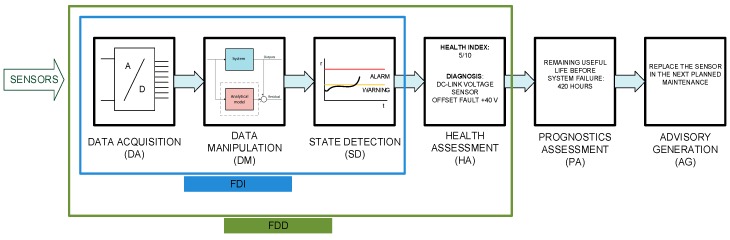
Example of the ISO 13374 functionalities for a condition-based maintenance (CBM) in sensors.

Early fault detection and diagnosis (FDD) is compulsory for a cost-effective and reliable CBM [3]. In contrast to Fault Detection and Isolation (FDI) strategies that are limited to fault detection and location, FDD also provides the severity of the fault [4], which can be important information for maintenance decision-making, in order to schedule the maintenance task, depending on the priority level and system degradation [5]. Both strategies are shown in the ISO 13374 framework in Figure 1. Due to limitations in train to remote supervision communications, on-board and online implementation is more suitable for State Detection functionality defined in ISO standard 13374. Furthermore, information related to severity and fault mode could be essential to get an optimal fault tolerant system, by means of parameters change or reconfigurations in an automatic way, for example by substituting the measured value by the estimated value [6]. 

Due to the importance of railway Traction Drive in the train availability, different FDI approaches have been implemented [7] in order to detect and isolate faults in sensors, electric machines and power converters. FDI approaches are mainly classified into model-based and data-driven techniques [8]. 

Model-based techniques need a knowledge of the physical system in order to build a model, which normally requires an important effort in complex systems, but as an advantage, it is suitable for on-board and real-time implementation, as it requires neither a large quantity of data, nor high computational resources. The model-based techniques can be divided into two parts: the residual generation and the residual evaluation. On the other hand, data-driven techniques do not need a knowledge of the physical system, but the computational burden is normally higher. Moreover, data quantity to process and storage is large, so it makes difficult its on-board implementation.

Because of the sensors’ contribution to traction control strategy, a sensor failure leads to a loss of availability of the railway traction drive. Thus, fault diagnosis approaches have been implemented in current, voltage and speed sensors in railway traction drive systems [9,10,11,12]. In most of them, model-based techniques have been utilized, although data-driven techniques have been presented too [13]. Luenberger (LO) and sliding mode observer (SMO) are mainly used in model-based approaches. Comparisons among different observer-based FDI solutions have been presented [14,15]. Both LO and SMO have a lower computational burden than the Kalman filter (KF) for on-board implementation. The main advantage of SMO compared to LO is the robustness under parameter uncertainties, but it has the chattering due to discontinuous feedback gain as a disadvantage. Furthermore, SMO in combination with an equivalent injection signal [16,17] allows fault reconstruction, which provides essential information for a quick maintenance task or for a sensor reconfiguration. SMO-based sensor fault reconstruction has been presented in previous publications [18,19,20]. The majority of the research works are limited to FDI strategies [6,9], mainly for a substitution of the measured value within a fault tolerant strategy, without providing information related to fault type or fault severity. 

Thus, this research provides both the severity of the fault and the fault type (offset or gain), in order to get a more reliable decision making for maintenance tasks. First, the residual generation, given by the fault reconstruction, is online obtained by means of an SMO and fault injection signal, implemented in a commercial railway Traction Control Unit (TCU), which is part of a hardware-in-the-loop (HIL) platform. Then, the residual evaluation is carried out using the Generalized Likelihood Ratio (GLR) Test for a time window. The aim of the evaluation is to avoid the false detections due to transients or anomalies in the system. Once the fault is detected and presented as State Detection functionality defines in ISO 13374, the fault type is obtained via an offline procedure, based on the fault reconstruction and data from TCU. This procedure provides the sensor Health assessment defined in ISO 13374. Reliable information of the sensor fault allows a rapid setting of the sensor during the maintenance task in order to minimize the fault. Residual generation is implemented online, whereas the sensor Health Assessment is obtained offline, being executed on-board or in the remote supervision by sending the data.

The paper has the following structure: Section 2 presents the railway traction unit. In Section 3 the sensor fault diagnosis scheme is introduced. Section 4 presents the fault reconstruction for DC-link voltage and catenary current sensors. Section 5 proposes a fault evaluation based on the Generalized Likelihood Ratio. Section 6 shows the procedure to discriminate offset and gain faults in sensors. Finally, a discussion and conclusions are presented.

## 2. Railway Traction Unit Description and Input Filter Model

**Figure 2:**
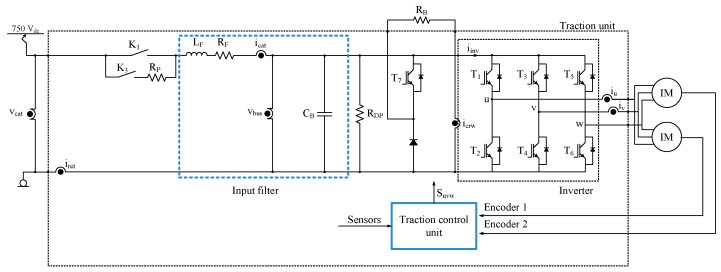
Railway traction unit.

Although there are different topologies for railway traction units, this research is focused on the traction unit shown in Figure 2. The sensors in the traction unit are summarized in Table 1. The current and voltage sensors are presented in Equation (1). A strategy for sensor fault reconstruction to DC-link voltage and catenary current sensors is applied, based on an SMO and the model of the input filter, presented in state space in Equation (1), being $x^{T}=\left[\begin{array}{cc} i_{cat} & v_{bus} \end{array}\right]$, $u^{T}=\left[\begin{array}{ccc} v_{cat} & i_{inv} & i_{crw} \end{array}\right]$ and $y^{T}=\left[\begin{array}{cc} i_{cat} & v_{bus} \end{array}\right]$. The $i_{inv}$ value is not directly measured, rather, it is calculated from T1, T3 and T5 switches states and $i_{u}$ and $i_{v}$ current sensors measurements.

The use of input filter has the advantage of providing a simpler model, which is not dependent on the motor parameters. Motor parameters are usually dependent on the operation point of the motor. Thus, the system only depends on the series inductance $L_{F}$, and the resistor $R_{F}$, and on the capacitor $C_{B}$, of the DC-link. Furthermore, the system based on the model of the input filter is suitable for DC-link voltage and catenary current estimations, and these sensors are the outputs of the proposed system.

(1) $$\begin{array}{c} \frac{dx}{\mathrm{dt}}=\left[\begin{array}{cc} -\frac{R_{F}}{L_{F}} & -\frac{1}{L_{F}}\\ \frac{1}{C_{B}} & 0 \end{array}\right]x+\left[\begin{array}{ccc} \frac{1}{L_{F}}\; & 0 & 0\\ 0 & -\frac{1}{C_{B}} & -\frac{1}{C_{B}} \end{array}\right]u\\ y=\left[\begin{array}{cc} 1 & 0\\ 0 & 1 \end{array}\right]x \end{array}$$

## 3. Sensor Fault Diagnosis Scheme

The proposed sensor fault diagnosis aims to get reliable information related to the fault mode, in order to carry out a maintenance task or a sensor reconfiguration. Thus, the severity of the fault, and its type, e.g. offset or gain faults, needed to be obtained. 

**Figure 3:**
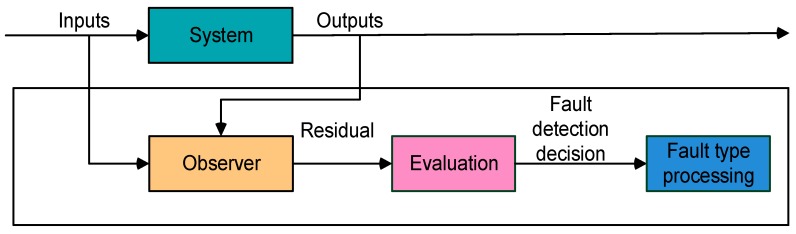
Proposed fault diagnosis scheme.

An observer-based fault diagnosis scheme is presented in Figure 3. The observer is used to obtain a residual, which should be close to zero in case of sensor fault-free cases. This residual, which is usually obtained from the difference between the estimated and the measured value, is compared to a threshold in order to detect and isolate the fault. Normally the residual is influenced by different faults, so a bank of observers is needed in order to isolate the fault [21]. 

Due to constraints in the communication to the remote maintenance center and the limitation for onboard data storage, observer-based approaches are more suitable for onboard implementation in the traction control unit. Furthermore, the lack of high-quality historical data with monitoring sample time short enough for analyzing signals with high dynamics, makes observer-based FDI more suitable than data-driven FDI. 

Among the observers, the LO is the most usual for industrial applications [22]. Slotine et al. presented the addition of a relay or saturation function to an LO as a way to provide robustness against uncertainties or disturbances, similar to the gain presented in an SMO [23]. Xia et al. mentioned the SMO to have better robustness to disturbance and lower sensitivity to parameter uncertainties than the LO [6]. 

In [24] several advanced observers are presented, and these observers allow implementing an FDD approach, as they are able to reconstruct the fault based upon the construction of an augmented system. Among them, Spurgeon previously presented the SMO [25], which reconstructs the fault based on the principle of equivalent injection signal. 

Although the LO is not discarded for this application, being the system lineal, (as it was previously tested in [12], due to the additional properties of the SMO, such as the possibility to provide the fault reconstruction and the robustness to disturbances), this observer will be analyzed.

Thus, based on the inhered disturbance presented in the catenary of a railway application, an FDD strategy based on an SMO for an augmented system has been selected. The fault reconstruction will be the residual for the evaluation.

The second step of the FDD strategy will be the evaluation. The evaluation is normally done by mean of norms for signals [26], statistical methods as the GLR [27] or machine learning [28], which needs the historical data for training. 

The minimum fault to detect for each sensor has been set based on residual values during dynamic simulation for sensor fault-free cases. The evaluation procedure does not need to be instantaneous, as no action will be taken before the next train stop, so it is offline implemented. The SMO is online executed, and it provides the fault reconstruction for a time window to the evaluation. In order to avoid the influence of disturbance or transients in the fault reconstruction during the evaluation, a GLR is applied to a time window of the reconstruction.

Finally, the fault type processing is done. The aim of this stage is to discern the type of fault, offset or gain, and the severity of the fault. The procedure to obtain the information is based on histograms which face the sensor fault reconstruction in a sensor to a measurement in other one. A processing of fault reconstructions and measurements is needed in order to eliminate the transients. 

## 4. Fault Reconstruction Based on Sliding Mode Observer and Input Filter Model

In this section the fault reconstruction based on an SMO for an augmented system is presented. The observer design and procedure to set the parameters is presented. Then the fault reconstruction for a different fault injected is simulated. Furthermore, the fault reconstruction is validated in a hardware-in-the-loop platform composed of a real time simulator and a commercial TCU for a tram.

### 4.1. Design of Sensor Fault Reconstruction Based on SMO

Garramiola et al. previously presented the sensor fault reconstruction for DC-link voltage and catenary current sensors [18], where the SMO parameter setting was widely explained. It is based on an SMO for an augmented system, where the sensor faults are treated as actuator faults. The fault reconstruction is obtained by the equivalent injection signal, understood as the average change in the feedback gain, in order to keep the difference among the measured and estimated values in the sliding surface, as it is shown in Equation (2).

(2) $${\hat{f}}_{i}={\left(A_{f}{}_{i}\right)}^{-1}v_{i}{}_{eq}$$

**Figure 4:**
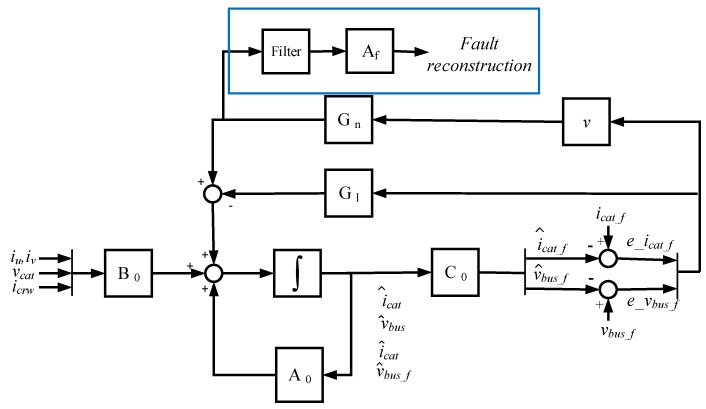
Sensor fault reconstruction based on the sliding mode observer (SMO).

The fault reconstruction under parameter variations, as well as under torque and catenary voltage changes, were analyzed and presented too. The SMO for the augmented system and the fault reconstruction based on an equivalent injection signal is presented in Figure 4.

The equations of the SMO are given in Equation (3). The SMO has two feedback matrix gains, shown in Equation (4), a linear gain given by the matrix G_l_ and a nonlinear gain based on matrix G_n_ and discontinuous term v, given by Equation (5), where p is a gain that should be higher than the maximum sum of sensor faults and uncertainties, in order to achieve the sliding motion, sat is the bound of the sliding surface and $e_{z}=\left[e_{z1}e_{z2}\right]=\hat{z}-z$, being $\hat{z}$ the estimated states and z the filtered measurements given by Equation (6), where $A_{f}$ is a positive, definite, diagonal matrix that represents inverse time constants [29]. The addition of the linear gain matrix G_l_ provides robustness against uncertainties in order to be stable [30]. Garramiola et al. previously presented the procedure to establish the SMO parameters [18]. First, the $A_{f}$ should be fixed, considering the dynamics of the dominant pole of the system. Then, the parameter Χ will be selected to set the observer dynamics. Finally, the sliding surface given by sat and the gain p should be calculated, in order to reach the sliding surface.

(3) $$\begin{array}{c} \left[\begin{array}{c} \dot{\hat{x}}\\ \dot{\hat{z}} \end{array}\right]=\underset{A_{0}}{\underbrace{\left[\begin{array}{cccc} -\frac{R_{F}}{L_{F}} & -\frac{1}{L_{F}}\; & 0 & 0\\ \frac{1}{C_{B}} & 0 & 0 & 0\\ A_{f1} & 0 & -A_{f1} & 0\\ 0 & A_{f2} & 0 & -A_{f2} \end{array}\right]}}\left[\begin{array}{c} \hat{x}\\ \hat{z} \end{array}\right]+\underset{B_{0}}{\underbrace{\left[\begin{array}{ccc} \frac{1}{L_{F}}\; & 0 & 0\\ 0 & -\frac{1}{C_{B}} & -\frac{1}{C_{B}}\\ 0 & 0 & 0\\ 0 & 0 & 0 \end{array}\right]}}u-G_{l}\left[\begin{array}{c} e_{z1}\\ e_{z2} \end{array}\right]+G_{n}\left[\begin{array}{c} v_{1}\\ v_{2} \end{array}\right]\\ \hat{z}=\underset{C_{0}}{\underbrace{\left[\begin{array}{cccc} 0 & 0 & 1 & 0\\ 0 & 0 & 0 & 1 \end{array}\right]}}\left[\begin{array}{c} \hat{x}\\ \hat{z} \end{array}\right]. \end{array}$$

(4) $$G_{l}=\left[\begin{array}{cc} 0 & 0\\ 0 & 0\\ 1 & 0\\ 0 & 1 \end{array}\right],\;G_{n}=\left[\begin{array}{cc} 0 & 0\\ 0 & 0\\ -A_{f1}+Χ & 0\\ 0 & -A_{f2}+Χ \end{array}\right].$$

(5) vi={−pAf(1sat)ezi−pAf(ezi|ezi|)          if ezi>sat or ezi<−satif sat> ezi>−sat,

(6) $$\dot{z}=-A_{f}z+A_{f}y.$$

### 4.2. Simulation of Fault Reconstructions for DC-Link Voltage and Catenary Current Sensors 

The SMO and the fault injection are modeled in Matlab-Simulink environment and tested in simulation, analyzing the following points:

- Dynamic response and robustness under torque and catenary voltage changes.
- Sensitivity of the fault reconstructions under variations of the input filter parameter
- Robustness under noise in sensor measurements

#### 4.2.1. Dynamic Response and Robustness Under Torque and Catenary Voltage Changes

**Figure 5:**
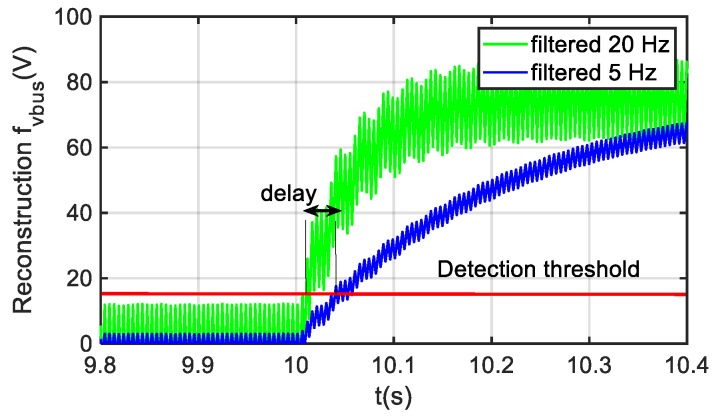
Fault reconstruction dynamics based of filter cut-off frequency.

The nonlinear gain of SMO, in this case based on a saturation function, allows a higher gain than the linear gain of an LO within a sliding surface, without increasing the sensibility to transients or disturbances. This fact could provide a faster response, but in the SMO solution proposed, the generation of the augmented system introduces a delay in the new state variable z given by Equation (6) due to the filtering given by $A_{f}$. Furthermore, due to the filtering of the fault reconstruction shown in Figure 4 an additional delay is included. The delay depends on the cut-off frequency of the filter, as it is presented in Figure 5. The fault reconstruction has a ripple due to the chattering effect of nonlinear gain, so a balance between ripple and dynamic response should be taken. For this application, the time for detection was not critical, so a cutoff frequency of 5 Hz could be taken in order to reduce the ripple of the fault reconstruction.

**Figure 6:**
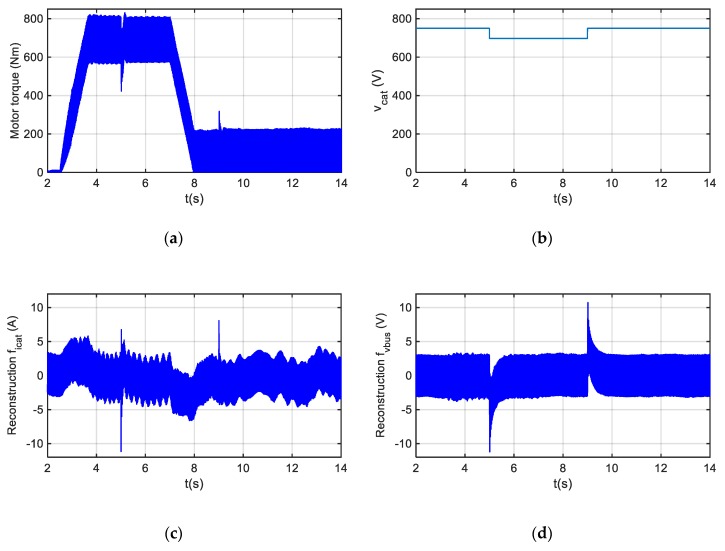
Fault reconstructions for a sensor fault-free scenario with a catenary voltage drop. (**a**) Motor torque. (**b**) Catenary voltage. (**c**) Catenary current sensor fault reconstruction. (**d**) DC-link voltage sensor fault reconstruction.

**Figure 7:**
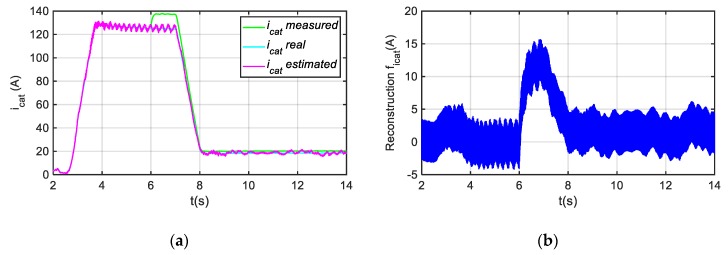
Injection of a gain fault of +10% in catenary current sensor. (**a**) Measure, real and estimated catenary current. (**b**) Catenary current sensor fault reconstruction.

In Figure 6, the dynamic response in case of a catenary voltage drop from 750 V to 700 V is presented. The DC-link voltage sensor fault reconstruction reaches the steady state in 0.5 s. Thus, during the evaluation, the analysis of longer time windows is recommended in order to minimize the false detections. On the other hand, in Figure 7, a gain fault of +10% is injected in the catenary current sensor in the instant in which t = 6 s. The catenary current depends upon the motor operation point. Thus, the amplitude of the fault reconstruction decreases from instant t = 7 s, as the motor torque reference decreases, and in consequence the catenary current too. 

#### 4.2.2. Sensitivity of the Fault Reconstructions under Variations of the Input Filter Parameter

The observers have been implemented for the filter model, which is composed of the series resistor and inductance and the DC-link capacitor. An analysis of fault diagnosis in steady state under the variation of these parameters has been done. Reasonable variations for series resistor (R_F_) and inductance (L_F_) are between ± 100% of the nominal value, whereas for DC-link capacitor (C_F_) variation goes from +100% to −50%, as a minimum capacity is needed in order to work. 

Only for a variation in the series resistor value, a significative change in the fault reconstruction for DC-link voltage is given. The maximum deviation of the residual is given for a variability of +100% of the resistor value and maximum torque, being the deviation equal to 8.22 V, as it is shown in Table 2. Thus, it is not recommended to establish a detection threshold under this value, otherwise a false positive could be given.

#### 4.2.3. Robustness under Noise in Sensor Measurements

**Figure 8:**
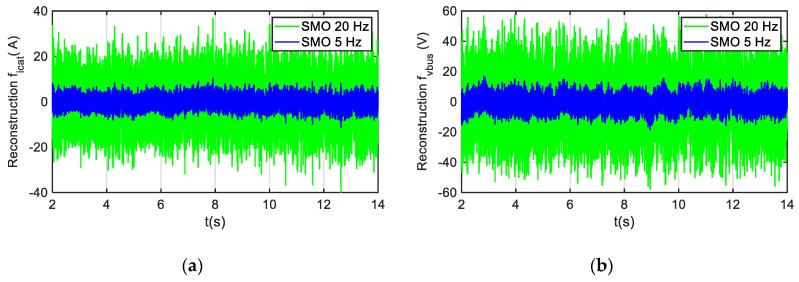
Residuals under noise in sensor measurements. The noise injected is uniformly distributed random noise with a frequency of 1 kHz. (**a**) Residuals for catenary current sensor. (**b**) Residuals for DC-link voltage sensor.

Analyzing the robustness under noise in sensor measurements, the SMO has presented robustness under uniformly distributed random noises. Since the fault reconstruction in the SMO is based on the new state variables, which are a filtered version of original ones, the SMO-based solution is less sensitive to noises. Thus, it makes that the average value of the fault reconstruction is not affected in steady state for a fault-free scenario. In Figure 8, the evolution of the residuals for both sensors are presented for two different filterings. Although the ripple of the fault reconstructions does increase due to injected noise, the average value of both reconstructions is around zero for a fault-free case. As the following evaluation will be based on average values for a time window, the ripple is not critical for this application.

### 4.3. Hardware-in-the-Loop Validation 

**Figure 9:**
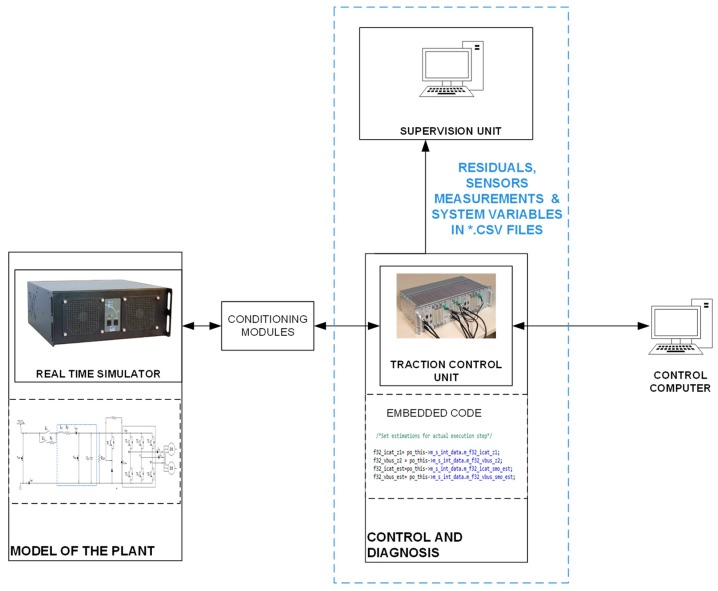
Hardware in the loop platform.

Then, the FDD strategy is implemented in a commercial TCU and tested in an HIL platform. This HIL platform is composed of a real time simulator where the traction unit plant is modeled, and a commercial TCU for a tram application, as it is shown in Figure 9.

In order to validate the FDD strategy, HIL simulations have been carried out for different operation points of the motor, as it is shown in Table 3. Several offset and gain faults have been injected into the DC-link voltage and catenary current sensors. Simultaneous faults have been injected too. 

**Figure 10:**
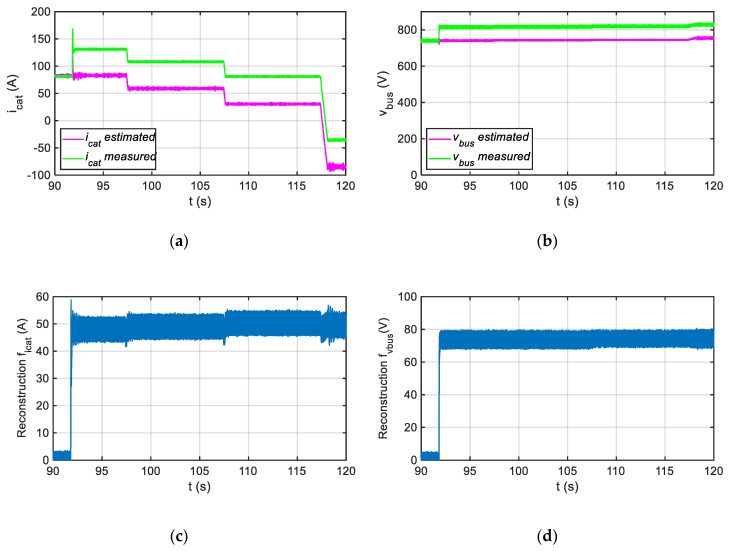
Injection of multiple sensor faults. (**a**) Measured and estimated catenary current. (**b**) Measured and estimated DC-link voltage. (**c**) Catenary current fault reconstruction. (**d**) DC-link voltage fault reconstruction.

The solution proposed is able to provide the fault reconstructions for both sensors, the DC-link voltage and catenary current, even if the faults in both sensors are injected simultaneously, as it is shown in Figure 10, where offset faults of +75 V and +50 A are injected in the instant that t = 91.8 s in the DC-link voltage and catenary current sensors, respectively.

**Figure 11:**
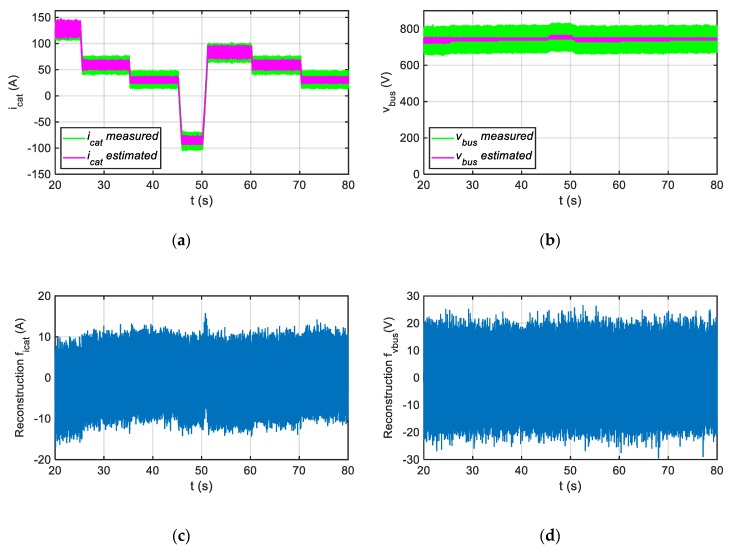
Injection noise in sensor measurements. The noise injected is uniformly distributed random noise with a frequency of 1 kHz. (**a**) Measured and estimated catenary current. (**b**) Measured and estimated DC-link voltage. (**c**) Catenary current fault reconstruction. (**d**) DC-link voltage fault reconstruction.

Furthermore, as it is shown in Figure 11, the average value of the sensor fault reconstructions is not significantly affected by the injection of noise in the sensor measurements. Thus, for a fault free case, the average values of the DC-link voltage and the catenary current sensors fault reconstructions are 0.86 V and 0.64 A, which it is not significant for a traction drive application.

The average values of the deviation between the fault injected and fault reconstruction for some of the most significative HIL simulations are shown in Table 4. The deviation is calculated as $e_{ficat}=f_{icat}-{\hat{f}}_{icat}$ for the catenary current sensor and $e_{fvbus}=f_{vbus}-{\hat{f}}_{vbus}$ for the DC-link voltage sensor, with $\hat{f}$ representing the fault reconstruction. It should be mentioned that in all the cases analyzed, the absolute value of $e_{ficat}$ remains under 4 A, and the absolute value $e_{fvbus}$ under 2 V.

The fault reconstructions, sensor measurements and system variables, such as torque estimation, are sent in a file system to an external supervision unit, through Ethernet-based communication, for further analysis. In a real application, the data analysis can be done on-board in the supervision unit, and engineers could just send the Health Assessment information to the remote supervision for maintenance decision-making, or send the files including the monitored data for analysis in the remote supervision, which could suppose a delay in the decision-making due to train-to-ground communications constraints.

## 5. Fault Evaluation based on Generalized Likelihood Ratio

Among the statistical approaches for fault evaluation, Ding presented the Generalized Likelihood Ratio (GLR) [31]. The GLR is based on the Likelihood Ratio (LR), which is based on the relation between two probability densities for a sample i of the signal r, one of probability densities for a healthy case and the other one for a faulty case, as it is shown in Equation (7). In case of a signal of n samples, the LR is given by Equation (8). The probability density for the sample i of the signal r to belong to a normal distribution, with mean value θ and variance $\sigma^{2}$, is shown in Equation (9). The main advantage of GLR compared to LR [31] is that it is not needed to calculate the probability density, and the maximum likelihood ratio is estimated from the average value of the signal $\bar{r}$ for n samples, as it is shown in Equation (10). Thus, the computational requirements are decreased.

(7) $$s_{i}\left(\theta_{1},\theta_{0}\right)=\ln\frac{p_{\theta_{1}}\left(r_{i}\;\right)}{p_{\theta }{}_{0}\;\left(r_{i}\right)}$$

(8) $$S\left(r\right)=\overset{n}{\underset{i=1}{\sum }}s_{i}\left(\theta_{1},\theta_{0}\right)$$

(9) $$p_{\theta }\left(r_{i}\;\right)=\frac{1}{\sigma \sqrt{2\pi }}e^{-\frac{{\left(r_{i}-\theta \right)}^{2}}{2\sigma^{2}}}$$

(10) $$\hat{S}\left(r\right)=\frac{n}{2\sigma^{2}}\left({\bar{r}}^{2}\right)$$

The GLR is applied to the residual given by the fault reconstruction shown in Figure 4, for a time window of n samples, in order to evaluate the faults in the DC-link voltage and catenary current sensors. Thus, these residuals are close zero in the case of a healthy state. The threshold for both sensors is set based on the minimum fault to detect, and taking into account that the threshold should be over the maximum value of the GLR for the different residuals, obtained in sensor fault-free simulations, as the false positive ratio should be minimized. Furthermore, the minimum fault to detect is set based on the quantitative effects of the sensor fault in the system, obtained by means of fault injections and dynamic simulation [32], which provides the maximum values of residuals for sensor fault-free scenarios. 

In conclusion, GLR calculation provides robustness to transients, due to changes in the motor operation point, or in the catenary voltage and noise in measurements, as it is based on the average value of the residual for *n* samples. It decreases the number of false detections compared to the usual evaluations, where each residual sample is compared to a threshold set by a norm-based method. Moreover, the dynamic simulation for fault-free scenarios allows obtaining the maximum value of the residual for fault-free scenarios, which will be used for the threshold calculation for GLR evaluation; the minimum threshold should be over the value of GLR obtained for fault-free scenarios. 

**Figure 12:**
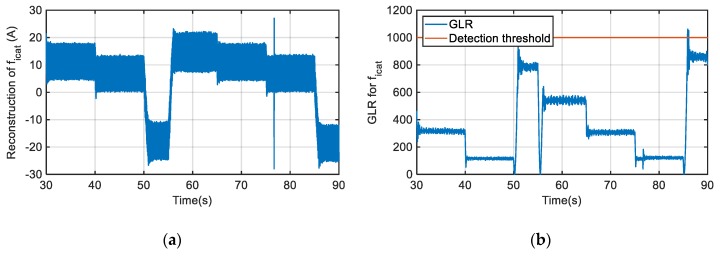
Gain fault injection in catenary current sensor. (**a**) Catenary current sensor fault reconstruction. (**b**) Generalized Likelihood Ratio (GLR) calculation.

In Figure 12 the fault reconstruction and the GLR calculation for a gain fault of +20% injected in the catenary current sensor, are shown. For GLR and threshold calculation time, windows of 500 samples have been used. The threshold has been set based on Equation (10) to detect a minimum sensor fault equal to 20 A. The fault reconstruction value depends on the operation point, as a gain fault has been injected, but only during the last braking period in t = 86 s overpasses the threshold set for GLR calculation for several samples.

Moreover, the fault reconstruction can be affected by operation transients, such as torque change or catenary voltage variation, as well as by the chattering effect due to a nonlinear feedback gain of SMO, or noises in the sensor measurements. In order to minimize theses effects, average values for a time window are utilized. A time window of 500 samples, being the monitoring sample time equal to 120 μs*,* allows us to get the average value of fault reconstruction and sensor measurements for a window of 60 ms, which is enough to minimize the aforementioned effects. Thus, as it can see in Figure 12, a transient in the fault reconstruction in the instant that t = 76.7 s is given due to a catenary voltage decrease of 40 V, but the GLR calculation does not overpass the threshold, as it is based on the average values of fault reconstruction for a time window.

Meinguet et al. used the GLR to improve the robustness of the fault detection and isolation (FDI), applying a cumulative sum (CUSUM) algorithm to several samples of the signal [33]. The threshold $J_{th}$ is given by Equation (11), being $f_{min}$ the minimum sensor fault to detect, $\sigma^{2}$, the variance of the fault reconstruction, which it is assumed to be constant, $T_{s}$ the monitoring sample time of the signal, and $t_{detection}$, the time within the fault should be detected, otherwise the cumulative sum is initialized.

Thus, it is not enough to overpass the threshold once to accept the faulty state. An alternative way could be to fix a counter, so the threshold should be overpassed for several times to accept the faulty state. The advantage of the CUSUM algorithm compared to the counter, is that the detection speed is linked to the fault severity, being quicker for higher faults.

(11) $$J_{th}=\frac{1}{2}\frac{{\left(f_{min}\right)}^{2}}{\sigma^{2}}\cdot t_{detection}\cdot \frac{1}{T_{s}}$$

The CUSUM GLR is presented in Equation (12) in case of $g_{k-1}>0$ and ${\hat{S}}_{k}>0$, otherwise $g_{k}\left(\theta_{1},\theta_{0}\right)=0$, being ${\hat{S}}_{k}$ obtained from Equation (10). The maximum value for the index k, being k∈Ζ, is given by the relation between $t_{detection}$ and $T_{s}$, and that for the aforementioned values is k=33.

(12) $$g_{k}\left(\theta_{1},\theta_{0}\right)=\max\left(0,\;g_{k-1}+{\hat{S}}_{k}\right)$$

**Figure 13:**
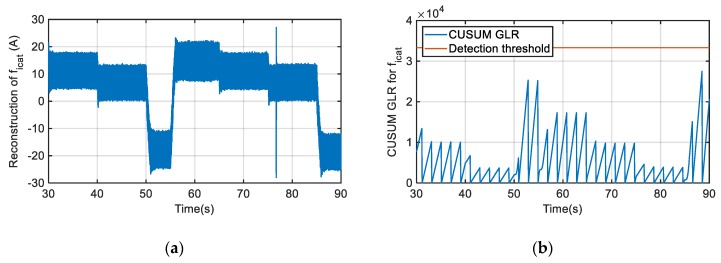
Gain fault injection in catenary current sensor. (**a**) Catenary current sensor fault reconstruction. (**b**) cumulative sum (CUSUM) GLR calculation.

In Figure 13 the previous gain fault case for the catenary current sensor is again presented, but in this case the CUSUM GLR is calculated. As the fault reconstruction does not overpass the minimum fault to be detected continuously, it is not enough to overpass the threshold within the detection time, which has been set to 2 s. Once the detection time has expired, the CUSUM GLR is restarted.

**Figure 14:**
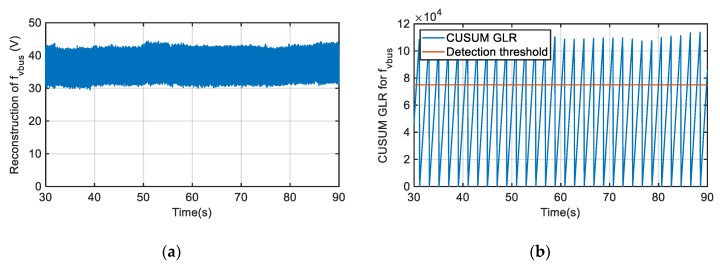
Gain fault injection in DC-link voltage sensor. (**a**) DC-link voltage sensor fault reconstruction. (**b**) CUSUM GLR calculation.

In a similar way, the same CUSUM GLR calculation can be applied for a gain fault injected in the DC-link voltage sensor. The minimum DC-link voltage sensor fault to be detected is set to 30 V. As it can be seen on Figure 14, for an injected gain fault of +5%, the threshold is permanently overpassed within the detection time, which has been set to 2 s again. 

**Figure 15:**
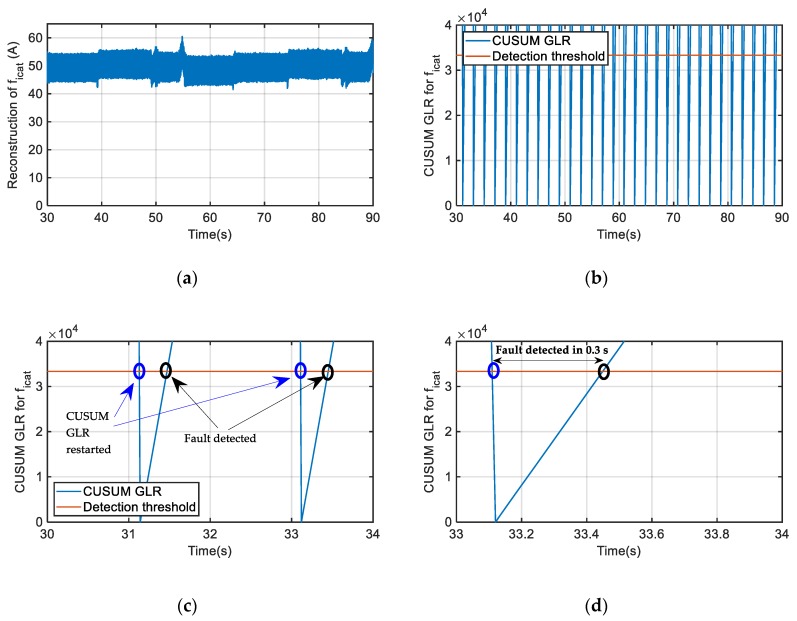
Offset fault injection in catenary current sensor. (**a**) Catenary current sensor fault reconstruction. (**b**) CUSUM GLR calculation. (**c**) Zoom of CUSUM GLR. (**d**) Time for fault detection.

Another case for the catenary current sensor is presented in Figure 15. An offset fault equal to +50 A is injected in the catenary current sensor measurement. As the fault injected is always over the minimum fault to be detected, set to 20 A, the CUSUM GLR calculation always overpasses the threshold within the detection time. The CUSUM GLR calculation is restarted every 2 s, as Figure 15c shows. In this case, the fault detection approximately takes 0.3 s, as it is shown in Figure 15d. As previously mentioned, the higher the fault, the shorter the time for fault detection.

**Figure 16:**
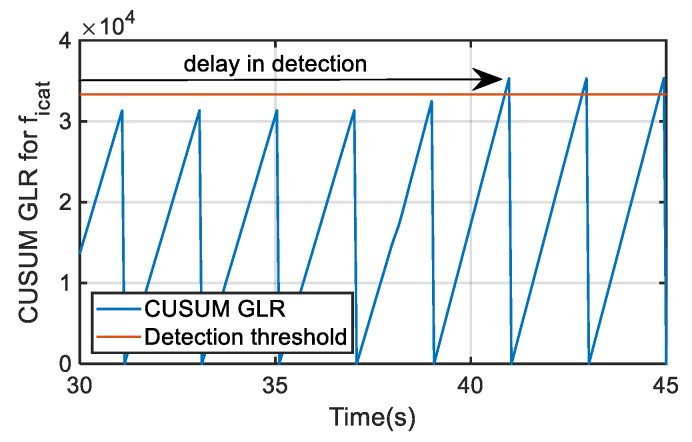
CUSUM GLR calculation for a critical offset fault injection in catenary current sensor.

In the case that the injected offset fault is decreased to +20 A, which is a critical value around the minimum fault to detect, the time needed for detection should be close to the 2 s set for detection time, but it could be longer, depending on the fault reconstruction average value, which is slightly influenced by the operating point. Thus, for catenary current fault reconstruction data during 15 s and the offset fault permanently injected, only in some cases the CUSUM GLR calculation overpasses the threshold, and in consequence, the fault is detected within the detection time. Thus, this can suppose a time delay in the fault detection, as it is not detected in every time window, as it is shown in Figure 16. Once the threshold is overpassed, a logical fault indicator is activated, indicating the presence of a fault in the corresponding sensor. The key points of the fault evaluation are the robustness of the detection and the absence of false positives, so a delay in the detection is not critical.

## 6. Procedure to Discern between Offset and Gain Faults 

Finally, once the fault generation and evaluation have been implemented and the fault decision is taken, the next step is the definition of the suitable maintenance action. The final goal of the procedure is to assess that there is a quasi-constant value of gain or offset fault in order to first evaluate the impact of this fault on the full performance of the traction drive (maximum efforts, efficiency, comfort, ..) and secondly, in the most unfavorable cases, in order to try to calibrate the sensors in an automatic reconfiguration procedure, during the short time stop in one station of the train.

For this point, and based on Health Assessment functionality defined in the ISO 13374 framework, and shown in Figure 1, the information related to the fault mode should be as complete as possible. The aforementioned FDD technique allows us to detect and isolate the sensor fault. Furthermore, due to equivalent injection signal, it is possible to obtain the fault reconstruction. Despite this information, in the case of gain faults, the fault reconstruction is changing depending upon the operation point, so the fault reconstruction is not enough for a quick and reliable definition of a maintenance task. Thus, in this section, an automatic procedure to discern between gain and offset sensor faults is presented. 

**Figure 17:**
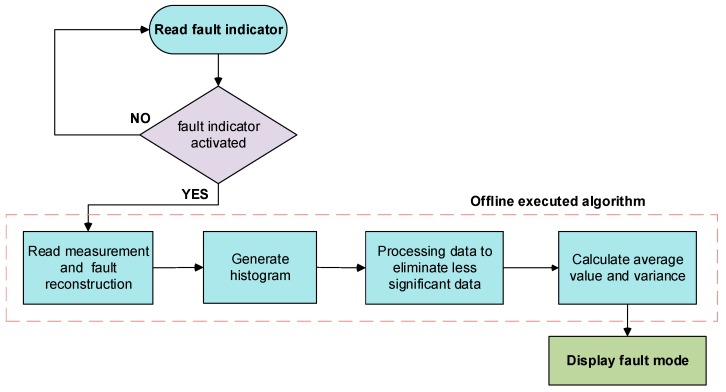
Flowchart for the procedure to discern gain and offset faults.

The procedure requires sensor measurements and fault reconstructions, and it is offline executed. Thus, in the case of a DC-link sensor fault, the DC-link voltage sensor fault reconstruction and the catenary current sensor measurement are needed. On the other hand, for the catenary current sensor, its fault reconstruction and the DC-link voltage sensor measurement are utilized. It is important to remark that the data collected should correspond to an operating profile with different torques, in order to ensure a variability in the current and voltage measurements. The procedure is summarized in Figure 17.

The procedure, suitable for DC-link voltage and catenary current sensors, can be divided into the following steps:

1. Read the sensor fault indicator, and in case the indicator is activated, keep on the next step. Otherwise, stay in this point.
2. Read the sensor measurements and fault reconstructions for a period of time, no less than one minute. During the time windows a variation in DC-link voltage and catenary current is needed in order to discern between gain and offset faults.
3. Calculate average values of the data for n samples, for example 500 samples, in order to minimize the effect of transient and measurement noises. The new vectors composed of average values will be used for the histogram.
4. Generate a 2D histogram with the vectors of average values. In the case of Health Assessment for a DC-link voltage sensor, in the x axis the catenary current measurement vector is represented, and in the y axis the DC-link voltage sensor fault reconstruction vector. On the other hand, for the catenary current sensor, in the x axis, the DC-link voltage measurement vector is represented, and in the y axis the catenary current sensor fault reconstruction vector.
5. Calculate the sum of the number of points for each interval of x vector (measurement data), and for each interval of y (fault reconstruction). The increment of the interval in both axes is equal to 1. If the sum of the number of points for each interval of x or y is less than the 2% of the total points, the value of the sum of the corresponding axis interval are substituted by zero, generating a new vector for both axes. This allows eliminating transients in fault reconstructions and measurements.
6. Calculate the percentage of the sum of points, referred to the total data points, for each fault reconstruction interval of the new vector (y axis), and then eliminate the fault reconstruction interval values, which percentage is less than 5%. This makes easier the automation of average value and the variance calculation of the resulting fault reconstruction vector.
7. Calculate the average value and variance of the fault reconstruction vector obtained in Step 6. The variance is compared to a threshold, set based on the variability of the fault reconstruction for different operating points. If the variance is higher than the threshold, then the fault mode is a gain fault, otherwise it is an offset gain. Based on the fault mode, the fault reconstruction and the measurement, the severity of the gain, or the offset fault, is calculated.

The histogram represents the relation between the fault reconstruction for a sensor and the measurement of another sensor. Thus, the fault reconstruction for the DC-link voltage sensor is faced with the catenary current measurement. In the case that the fault reconstruction remains constant for a variable catenary current measurement, we can conclude that there is an offset fault in the DC-link voltage sensor, otherwise a gain fault is assumed. A previous processing of both signals is needed in order to discard transients in signals. On the other hand, the fault reconstruction for the catenary current sensor is faced with the DC-link voltage measurement. This procedure needs a variation in the sensor measurements to work properly, so if there is not any variation in the measurement, a new period should be analyzed for decision making.

**Figure 18:**
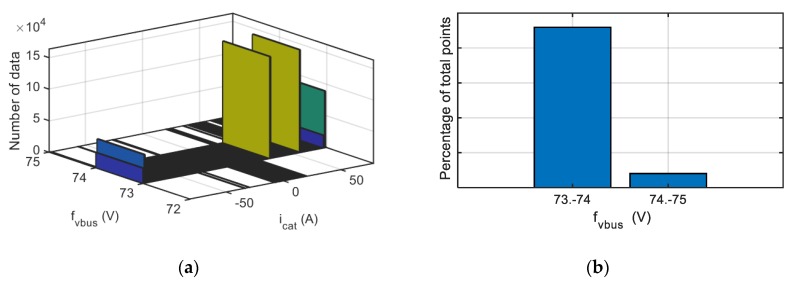
Visualization for an offset fault injected in DC-link voltage sensor. (**a**) Histogram. (**b**) Percentage of points for each fault reconstruction interval.

In Figure 18 the results of the procedure for an offset fault injection in the DC-link voltage sensor is shown. As it can see in Figure 18a, the histogram is defined from the DC-link voltage sensor fault reconstruction and catenary current measurement, then the data is processed, being the result of Step 6 displayed on Figure 18b. The algorithm provides an offset fault with an average value of 73.66 V, which makes easier the fitting of the sensor during a maintenance action or an automatic reconfiguration of the sensor.

**Figure 19:**
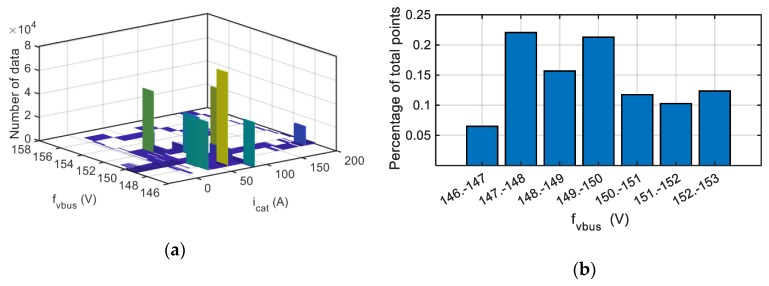
Visualization for a gain fault injected in the DC-link voltage sensor. (**a**) Histogram. (**b**) Percentage of points for each fault reconstruction interval.

In Figure 19, the same procedure is applied for a gain fault injection equal to +20% in the DC-link voltage sensor. The algorithm provides a gain fault in the DC-link voltage sensor, being the gain of the sensor calculated 1.199 instead of the nominal gain equal to 1. This information allows a quick maintenance action or a reconfiguration of the sensor.

**Figure 20:**
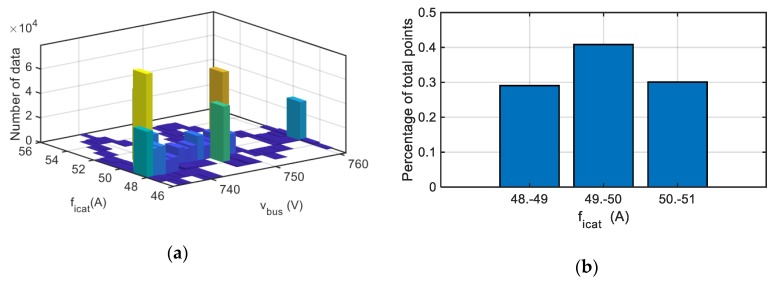
Visualization for an offset fault injected in catenary current sensor. (**a**) Histogram. (**b**) Percentage of points for each fault reconstruction interval.

In a similar way, the algorithm is applied to the catenary current sensor, presenting the results for an offset fault equal to 50 A in Figure 20. In this case, the algorithm displayed an offset fault with a severity of 49.50 A.

**Figure 21:**
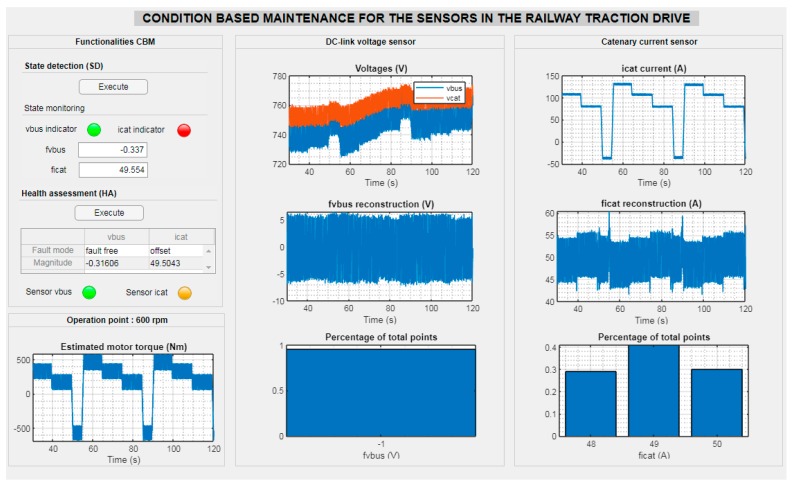
Interface for sensor Health Assessment for an offset fault of 50 A injected in catenary current sensor.

Although the fault and the Health Assessment is automatically done, in order to improve the reliability of the fault mode automatically obtained, additional information could be displayed in an interface, as it is shown in Figure 21 for an offset fault of +50 A injected in the catenary current sensor. Thus, maintenance experts could verify the sensor Health Assessment with visual information, and confirm the suitable maintenance action; e.g., do nothing, try an automatic sensor reconfiguration or plan a sensor repair/substitution. Apart from the fault type and magnitude obtained in the previous procedure presented in Figure 17, the DC-link and catenary voltage sensors and catenary current sensor measurements are displayed, as well as the sensor fault reconstructions for both sensors. Furthermore, in the Health Assessment a lamp is displayed, in which each color represents a severity level of the effects in the system for the sensor fault.

## 7. Discussion

In this article, a sensor fault diagnosis strategy has been presented. The strategy aims to get detailed and quantitative information related to the fault mode, which allows a quick maintenance action or an automatic reconfiguration of the sensor, in order to minimize the effects of the fault in the system.

First, an FDD technique based on an SMO and the equivalent injection signal has been presented. This FDD technique is able to detect and isolate the faulty sensor, as well as to provide the severity of the fault. The FDD technique has been implemented in an HIL platform composed of a real time simulator and a commercial railway TCU corresponding to a tram application. In order to increase the robustness of the fault decision, a fault evaluation based on a CUSUM GLR has been implemented. Although the implementation of the CUSUM GLR supposes a delay in the fault detection, the delay in the fault detection is not critical, being the priority of this research to avoid false positives. A reasonable time for detection could be the time between two stops. On the other hand, the evaluation minimizes the effects of chattering, noises and transients in measurements due to system dynamics, thus improving the robustness of the decision. 

Then, an algorithm to discern between offset and gain sensor faults has been presented. The FDD technique is not enough to provide the sensor Health assessment for a CBM. Thus, additional information related to the fault mode is needed. The algorithm is offline executed, based on fault reconstructions and sensor measurements for a certain period of time, for example several minutes, in order to ensure that there are variations of the traction drive operation point. The algorithm is offline executed, and it provides the fault type, offset or gain, and its severity. Based on this information and the effects of the fault mode in the system, a reliable maintenance action could be defined. Depending on the severity of the fault, the maintenance action could be immediately executed, planned or rejected. Maintenance action could be implemented in manual or automatic mode. 

The goal of this research is to provide reliable tools for a CBM to the supervision system, in order to prepare reliable maintenance actions in advance, which could reduce the maintenance costs in railway applications.

## 8. Conclusions

In this article a hybrid sensor fault diagnosis has been proposed. The strategy is able to detect and isolate the sensor fault, as well as being able to discern between offset and gain faults. Furthermore, due to SMO and equivalent injection signal, it is possible to get the severity of the gain or offset fault. A fusion of data from a model-based FDD, implemented in a commercial TCU and an HIL platform, and data from sensors, is used for Sensor Health Assessment. This information is fundamental for a reliable and quick maintenance action. Thus, this sensor fault diagnosis is in frame with a CBM strategy. 

## Figures and Tables

**Table 1:** Current and voltage sensors in the Traction Control Unit (TCU).

Sensor	Description
*v_cat_*	Catenary voltage sensors
*i_cat_*	Catenary current sensor
*i_ret_*	Return current to catenary sensor
*v_bus_*	DC-link voltage sensor
*i_crw_*	Braking unit current sensor
*i_u,v_*	Motor phase current sensors

**Table 2:** Analysis of fault reconstructions in steady state for a sensor fault-free scenario and parameter variation.

Parameter Variation.	$\mathrm{Reconstruction}\;f_{icat}$	$\mathrm{Reconstruction}\;f_{vbus}$
R = R_F_, L = L_F,_ C = C_F_	−0.31 A	−0.11 V
R = 2R_F_, L = L_F,_ C = C_F_	−0.51 A	−8.22 V
R = 0, L = L_F,_ C = C_F_	−0.35 A	7.88 V
R = R_F_, L = 2L_F,_ C = C_F_	−0.38 A	−0.11 V
R = R_F_, L = 0_,_ C = C_F_	−0.30 A	−0.10 V
R = R_F_, L = L_F,_ C = 2C_F_	−0.32 A	−0.10 V
R = R_F_, L = L_F,_ C = 0.5C_F_	−0.32 A	−0.10 V

**Table 3:** Summary of hardware-in-the-loop (HIL) validation and case studies.

Motor Torque Range	Offset Fault Injected	Gain Fault Injected	Fault Injection	Others	Number of Case Studies
From 175 Nm to 690 Nm	± 20, ±50, ±75,±100.	±10%, ±20%	Single or multiple faults	Noise, catenary voltage change, parameter variability	> 500.

**Table 4:** Summary of deviation between fault injected and fault reconstruction average values.

Motor Torque	Fault Injected	$e_{fvbus}$	$e_{ficat}$
690 Nm	$f_{vbus}=75\;V$	1.053 V	3.126 A
460 Nm	$f_{vbus}=75\;V$	0.146 V	1.415 A
330 Nm	$f_{vbus}=75\;V$	0.585 V	0.410 A
175 Nm	$f_{vbus}=75\;V$	−1.606 V	−0.756 A
690 Nm	$f_{icat}=50\;A$	0.631 V	3.707 A
460 Nm	$f_{icat}=50\;A$	−0.144 V	2.118 A
330 Nm	$f_{icat}=50\;A$	0.159 V	1.086 A
175 Nm	$f_{icat}=50\;A$	0.009 V	−0.131 A
